# Promoting Health and Well-Being Through Mobile Health Technology (Roadmap 2.0) in Family Caregivers and Patients Undergoing Hematopoietic Stem Cell Transplantation: Protocol for the Development of a Mobile Randomized Controlled Trial

**DOI:** 10.2196/19288

**Published:** 2020-09-18

**Authors:** Michelle Rozwadowski, Manasa Dittakavi, Amanda Mazzoli, Afton L Hassett, Thomas Braun, Debra L Barton, Noelle Carlozzi, Srijan Sen, Muneesh Tewari, David A Hanauer, Sung Won Choi

**Affiliations:** 1 Department of Pediatrics Michigan Medicine University of Michigan Ann Arbor, MI United States; 2 Department of Anesthesia Michigan Medicine University of Michigan Ann Arbor, MI United States; 3 Department of Biostatistics School of Public Health University of Michigan Ann Arbor, MI United States; 4 School of Nursing University of Michigan Ann Arbor, MI United States; 5 Department of Physical Medicine and Rehabilitation Michigan Medicine University of Michigan Ann Arbor, MI United States; 6 Department of Psychiatry Michigan Medicine University of Michigan Ann Arbor, MI United States; 7 Department of Internal Medicine Michigan Medicine University of Michigan Ann Arbor, MI United States

**Keywords:** family caregivers, mobile health app, mHealth, randomized controlled trial, wearable wrist sensor, hematopoietic stem cell transplantation, HSCT

## Abstract

**Background:**

Cancer patients who undergo allogeneic hematopoietic stem cell transplantation are among the most medically fragile patient populations with extreme demands for caregivers. Indeed, with earlier hospital discharges, the demands placed on caregivers continue to intensify. Moreover, an increased number of allogeneic hematopoietic stem cell transplantations are being performed worldwide, and this expensive procedure has significant economic consequences. Thus, the health and well-being of family caregivers have attracted widespread attention. Mobile health technology has been shown to deliver flexible, and time- and cost-sparing interventions to support family caregivers across the care trajectory.

**Objective:**

This protocol aims to leverage technology to deliver a novel caregiver-facing mobile health intervention named Roadmap 2.0. We will evaluate the effectiveness of Roadmap 2.0 in family caregivers of patients undergoing hematopoietic stem cell transplantation.

**Methods:**

The Roadmap 2.0 intervention will consist of a mobile randomized trial comparing a positive psychology intervention arm with a control arm in family caregiver-patient dyads. The primary outcome will be caregiver health-related quality of life, as assessed by the PROMIS Global Health scale at day 120 post-transplant. Secondary outcomes will include other PROMIS caregiver- and patient-reported outcomes, including companionship, self-efficacy for managing symptoms, self-efficacy for managing daily activities, positive affect and well-being, sleep disturbance, depression, and anxiety. Semistructured qualitative interviews will be conducted among participants at the completion of the study. We will also measure objective physiological markers (eg, sleep, activity, heart rate) through wearable wrist sensors and health care utilization data through electronic health records.

**Results:**

We plan to enroll 166 family caregiver-patient dyads for the full data analysis. The study has received Institutional Review Board approval as well as Code Review and Information Assurance approval from our health information technology services. Owing to the COVID-19 pandemic, the study has been briefly put on hold. However, recruitment began in August 2020. We have converted all recruitment, enrollment, and onboarding processes to be conducted remotely through video telehealth. Consent will be obtained electronically through the Roadmap 2.0 app.

**Conclusions:**

This mobile randomized trial will determine if positive psychology-based activities delivered through mobile health technology can improve caregiver health-related quality of life over a 16-week study period. This study will provide additional data on the effects of wearable wrist sensors on caregiver and patient self-report outcomes.

**Trial Registration:**

ClinicalTrials.gov NCT04094844; https://www.clinicaltrials.gov/ct2/show/NCT04094844

**International Registered Report Identifier (IRRID):**

PRR1-10.2196/19288

## Introduction

Approximately 2.8 million people in the United States are currently providing unpaid, informal care to an adult with cancer [1,2]. With the growing number of cancer survivors, family caregivers represent a critical extension of the national health care system [3]. Caregiving tasks are time- and labor-intensive, and include multifaceted activities [4]. Unfortunately, these experiences may lead to significant physical, psychological, emotional, social, and financial burdens, along with deleterious health effects [5-7]. There is broad agreement that caregiving is challenging and stressful. This is perhaps most pronounced in caregivers of patients undergoing allogeneic hematopoietic stem cell transplantation (HSCT), also commonly known as blood and marrow transplantation, who must address the intense and persistent caregiving needs of some of the most critically ill cancer patients, which continue throughout a prolonged hospital stay, followed by close outpatient follow up over many months [8,9].

Given the high risks associated with HSCT, a dedicated caregiver is necessary and expected for at least the first 100 days post-transplant [10]. However, HSCT caregivers are often unprepared for this role; thus, it is not surprising that HSCT caregivers experience significant levels of anxiety and distress, especially during the peritransplant period [8,9]. Psychoeducation, skills training, and therapeutic counseling interventions have been shown to benefit caregiver health and well-being [11]. However, there remain major barriers in translating successful interventions to clinical practice, including limited understanding of the mechanism of action of an intervention, and need for expert trainers, intensive training, and monitoring [12]. Thus, interventions that are mechanism-focused, low-cost, and sustainable are needed [13].

The ability to capture the hazards of caregiving (ie, adverse physical and mental health consequences) [14] accurately and in real time has been limited by assessments that have traditionally relied on long-term recall and self-report of symptoms. Mobile health (mHealth) technology remains relatively new in the clinical research area, but is spreading quickly and its costs are declining [15], which may facilitate stronger partnerships between patients, family caregivers, and health care providers [16]. In particular, mHealth technology can serve as a platform for the delivery of multicomponent health interventions while capturing continuous, real-time measures via wearable sensors (eg, sleep, physical activity). The extreme HSCT setting [17] provides an ideal “model” to rigorously test an mHealth intervention owing to (1) the high level of engagement by HSCT caregivers; (2) intense and rapidly evolving caregiving needs of medically fragile patients; and (3) long hospital course followed by frequent outpatient follow up that allow for high-resolution data collection with minimal additional burden [18].

In HSCT, malignant cells are first destroyed by chemotherapy and/or radiation therapy, followed by infusion of compatible donor cells to alleviate profound hematologic toxicities (eg, neutropenia, anemia, and thrombocytopenia) [19]. The main complications, some of which are life-threatening, include mucositis, nausea, vomiting, diarrhea, infections, and bleeding. Therefore, a dedicated 24/7 caregiver is necessary and expected. Frequently, a caregiver is tasked with monitoring treatment side effects, managing the symptom burden, making treatment decisions, administering medications, and performing medical tasks (eg, central line care and dressing changes). After engraftment of donor cells, graft-versus-host disease may ensue, which may further result in prolonged immunosuppression, morbidity, and mortality [20-22]. As such, the HSCT trajectory may be long and unpredictable [23], which creates a complex and multifaceted caregiving process. Caregivers can easily become overwhelmed and must juggle multiple roles: (i) “interpreter” of medical information; (ii) “organizer” of medical appointments and juggling the needs of other family members; and (iii) “clinician” to assess and identify health changes in the patient [23].

**Figure 1:**
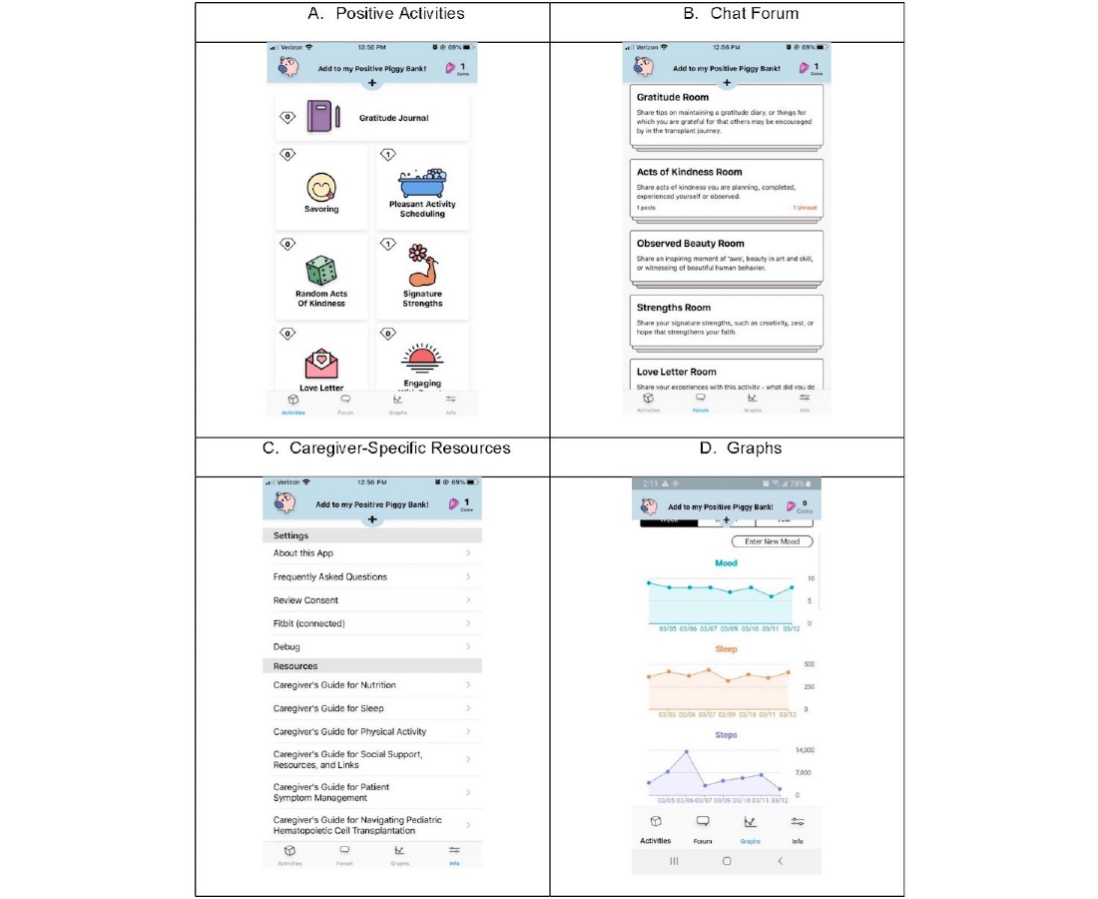
Screenshots of the Roadmap 2.0 app.

Our research team previously developed Roadmap 1.0 (formerly referred to as BMT Roadmap) as a caregiver-facing mHealth app. Details of the app with descriptions of each of the components have been published previously [24-26]. We iteratively designed and developed the Roadmap 2.0 mHealth app following the following 7 research phases: (1) Roadmap 1.0 prototype [27,28]; (2) design groups to evaluate the Roadmap 1.0 prototype [29]; (3) pilot study of Roadmap 1.0 [30-32]; (4) extensive literature review [33,34]; (5) design and development of Roadmap 2.0 in the outpatient setting [25]; (6) design and development of Roadmap 2.0 in the participant home setting [24]; and (7) deployment of a National Caregiver Health survey [25]. Figure 1 shows screenshots of key app features, and Multimedia Appendix 1 provides a detailed definition of the multicomponent features of the Roadmap 2.0 app.

Our preliminary data showed that family caregivers were interested in and willing to engage in positive psychology interventions to reduce stress and improve their health-related quality of life [18,19]. Herein, we provide a detailed description of the design for a mobile randomized controlled trial to test this investigator-initiated, multicomponent Roadmap 2.0 app comparing a positive psychology-based intervention arm with a control arm in HSCT family caregiver-patient dyads (the Roadmap 2.0 trial). We postulated that simple and intentional pleasant activities that could be developed into routine practices, such as expressing gratitude or scheduling activities that promote positive thoughts, emotions, or behaviors [35-37], may be effective and scalable in the HSCT caregiver population.

## Methods

### Study Design

#### Overview

**Figure 2:**
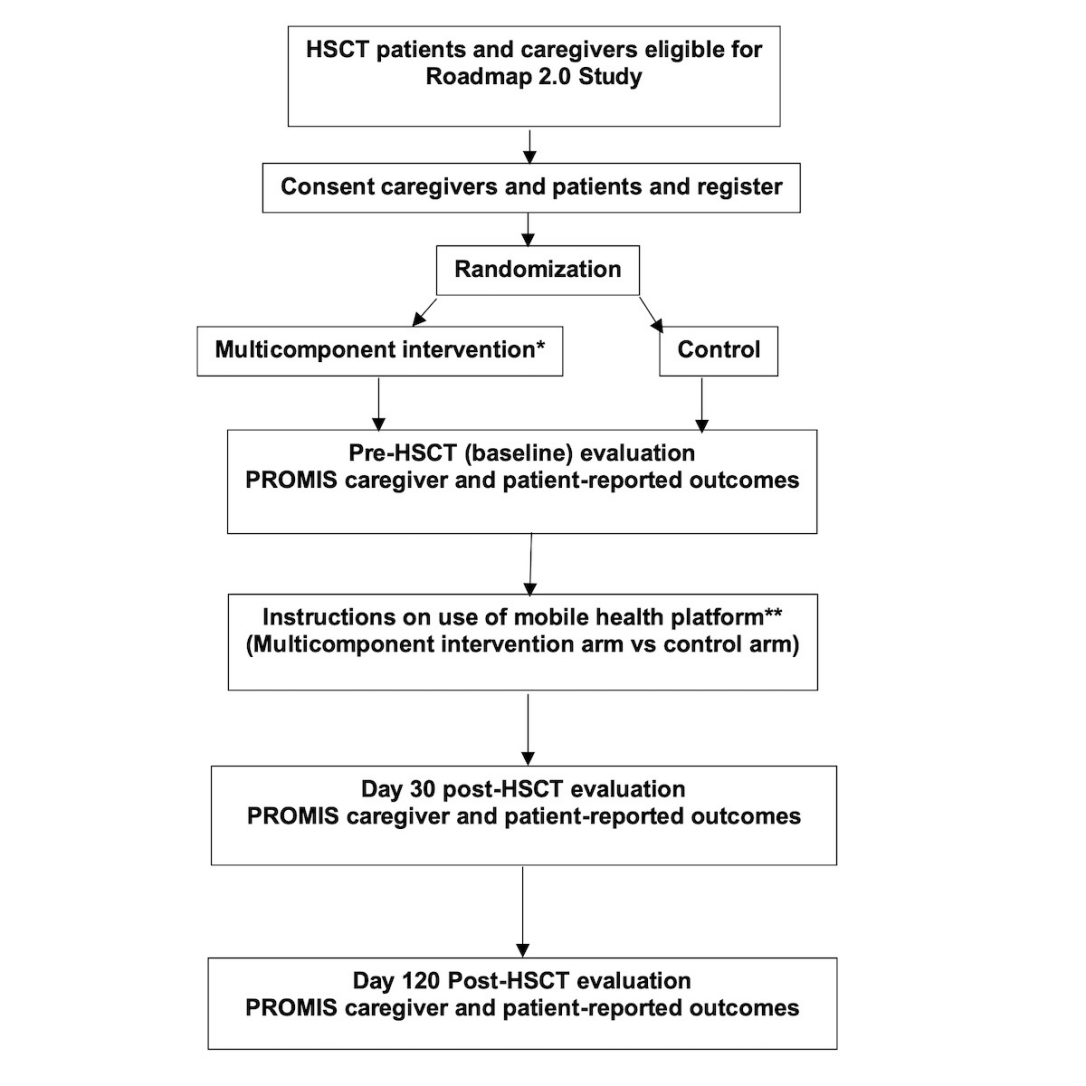
Study schema. HSCT: hematopoietic stem cell transplantation.

This study will test the effectiveness of a positive psychology intervention delivered via an mHealth technology platform (see Figure 2 for a schema of the study design). The trial is planned according to a two-arm randomized controlled design. Each of the caregiver-patient participants will be randomized to an active treatment arm (Roadmap 2.0 app + multicomponent features + Fitbit Charge 3) or to a control arm (Roadmap 2.0 app + Fitbit Charge 3; that is, the app displays physiological data only without any multicomponent features). The random allocation of participants to the treatment arm or control arm establishes the basis for testing the statistical significance or difference between the groups in the primary measured outcome (caregiver health-related quality of life, assessed by the Patient-Reported Outcomes Measurement Information System [PROMIS] Global Health scale [38]). Caregiver-patient age, gender, and other prognostic baseline characteristics that could potentially confound an observed association, including those that are unknown or unmeasured, will be distributed equally, except through chance alone, through random assignment. Thus, this design is well-suited to our goal of assessing the effectiveness of an mHealth intervention, Roadmap 2.0, on caregivers (Figure 2).

#### Primary Objective

The primary objective of this proposal is to test Roadmap 2.0 + multicomponent features in caregivers of patients undergoing HSCT. One half of the caregivers will be randomly assigned to the treatment arm and the other half will be assigned to the control arm.

#### Primary Endpoint

A single primary endpoint of caregiver health-related quality of life (as assessed by the PROMIS Global Health-10 scale [38]) at day 120 post-transplant is designated for the purpose of planning the sample size (see below) and duration of the study, and to avoid problems of interpreting tests of multiple hypotheses (for caregivers and patients).

#### Secondary Endpoints

Constructs of both caregiver and patient health-related quality of life include (1) positive aspects of caregiving, assessed by the PROMIS Companionship [39], PROMIS Self-Efficacy for Managing Symptoms [40], PROMIS Self-Efficacy for Managing Daily Activities [41], and Neuro-QoL Positive Affect and Well-Being scales [42]; (2) negative aspects of caregiving, assessed by the PROMIS Sleep Disturbance [43], PROMIS Depression [44], and PROMIS Anxiety scales [39]; and (3) other caregiver-specific well-being or health-related quality of life measures assessed by the TBI-CareQoL Caregiver Anxiety [45,46] and TBI-CareQol Caregiver Strain [45,47] scales (see Multimedia Appendix 2). Additional secondary endpoints will include both caregiver and patient health care utilization (eg, total count of hospital days, readmissions, and ambulatory care clinic visits within the first 120 days post-transplant) and patient infections, graft-versus-host disease, relapse, and survival.

#### Blinding

Study arm assignments cannot be blinded to the investigators because it will be known whether participants have Roadmap 2.0 on their mobile device or not (for technical purposes).

#### Intervention Period

The intervention will be delivered from the time of admission for the patient’s HSCT through day 120 post-transplant. Data will be collected preintervention (baseline), during the intervention (day 30 post-transplant), and at the end of the intervention (day 120 post-transplant). The length of the intervention is therefore approximately 16 weeks.

### Participant Enrollment and Evaluation

Participants (caregiver-patient dyads) will be approached for inclusion in the study after the decision to proceed with transplantation is made by the patient. Caregivers will be identified by the patient as their primary caregiver who will be providing at least 50% of the caregiving needs. Multiple caregivers are not allowed according to the study design. Participants willing to participate in this trial will sign an Institutional Review Board (IRB)-approved consent form. Transplant physicians will evaluate the patient eligibility to undergo HSCT as well as eligibility for randomization in this study. Eligibility criteria will be verified by the study team and the clinical trials support office. Ineligible participants will proceed off study and no further follow up will be obtained. The study team personnel (eg, research coordinator) will record the documentation of subject consent in the OnCore Clinical Trials Management System (a web-based system that supports all aspects of clinical research, including protocol management, patient registry, biospecimen repository, and budget tracking) and will be registered through the clinical trials support office.

The inclusion criteria for the caregiver are that they must have an eligible patient (see definition below) for whom they provide at least 50% of care needs, be at least 18 years old, able to read and speak English, and capable of signing an informed consent form. An eligible patient is one who: identifies the eligible caregiver as their primary caregiver (ie, provides at least 50% of their care needs), aged ≥18 years, scheduled to undergo HSCT, and able to read and speak English and to sign informed consent forms.

Patients and caregivers will agree to provide regulatory compliance and IRB-approved informed consent, in accordance with the Clinical Practice Guidelines of the University of Michigan Transplant Program. A patient is considered to be able to undergo HSCT at the University of Michigan only if a designated family caregiver (eg, parent, adult children, spouse, family member, neighbor, friend) accepts the roles outlined in the University of Michigan Caregiver Responsibility Contract, and both the caregiver and patient sign the contract. The exclusion criterion is therefore a patient who does not meet these eligibility criteria to undergo HSCT at the University of Michigan Transplant Program.

### Transplant Protocol Registration

Before randomization, the research coordinator must state the baseline caregiver biological characteristics (eg, age, gender, race/ethnicity) through the OnCore Clinical Trials Management System. This will avoid potential biases such as preferential use of gender in one arm of the study. At this stage, the research coordinator will also verify that the patient is still a candidate for transplantation, and that both the caregiver and patient are eligible for the trial.

### Randomization

Blocked randomization will be used to limit bias and achieve an equal distribution of participants to the control and treatment arms. Randomization will be overseen by the study statistician (TB).

Once the caregiver is deemed eligible and has provided written informed consent, the research coordinator will confirm their eligibility. The study statistician (TB) will create a randomization list before enrollment using the R statistical software package. The statistician will then forward the list to the clinical trials support office, who will be responsible for enrolling patients and assigning them to the correct study arm. Caregivers will not be randomized more than 21 days from the planned initiation of conditioning. All patient treatments related to the transplant will be scheduled *prior* to randomization. This includes planning an admission date and ensuring that donors can be used in a coordinated fashion with the planned transplant.

Participants (caregiver-patient dyad) randomized to the treatment arm (Roadmap 2.0 app + multicomponents features + Fitbit Charge 3) will be instructed on how to operate the Roadmap 2.0 app on their own mobile device and to create a password, and to use it freely throughout the intervention period. The caregiver and patient will receive their own respective passwords. Timestamps will be recorded of each participant’s use of Roadmap 2.0 (ie, number of logins and pages viewed, when and how long and in what order). Caregivers and patients will both be provided with Fitbit Charge 3 wearable wrist sensors.

Participants randomized to the control arm (Roadmap 2.0 app without any multicomponent features + Fitbit Charge 3) will be advised to use their own mobile device freely throughout the intervention period. Participants will receive usual care, defined as standard-of-care information provided according to the Clinical Practice Guidelines of the University of Michigan Transplant Program. This version of Roadmap 2.0 has no positive psychology-based activities, but caregivers and patients will both be provided with Fitbit Charge 3 wearable wrist sensors. Their view of the app will only include the “Settings” section but not the “Resources” section (Figure 1).

### Confidentiality

Confidentiality will be maintained by individual names being masked and each participant being assigned a patient identifier code. The code relaying the participant’s identity with the ID code will be kept separately at the Clinical Trials Support Office. The outcome measures will be deidentified and masked to the biostatistician/data scientist performing the analyses.

### Pretransplant Evaluations

Based on prior work, our study team has experience in obtaining caregiver demographics. The following data will be collected (ie, caregiver self-report) via the Roadmap 2.0 app within 30 days of randomization: demographic, caregiving, and personal health variables such as marital status, alcohol/tobacco use, education, employment, annual household income, use of technology, type of relationship to the patient, number of household members, health insurance status, number of years and number of hours per week (of caregiving), health comorbidities, medications, and caffeine intake.

The following data will be collected within 30 days of randomization, according to the Transplant Program Clinical Practice Guidelines in patients undergoing transplant: history, physical examination, height, weight, Karnofsky performance status [48], HSCT-specific Comorbidity Index Score [49], routine laboratory parameters (eg, complete blood count with differential and platelet count, serum creatinine bilirubin, alkaline phosphatase, aspartate transaminase, and alanine aminotransferase), infectious disease titers, electrocardiogram and left ventricle ejection fraction, pulmonary function tests, disease evaluation of underlying blood disease, chest X-ray or chest computed tomography, pregnancy test, pretransplant donor and recipient samples for post-transplant chimerism evaluation, and pretransplant blood samples for future research.

PROMIS caregiver and patient health-related quality of life assessments will be obtained at baseline, preintervention (Multimedia Appendix 2).

### Intervention

#### Overall Design

Participants (caregiver-patient dyad) who are consented, enrolled, and randomized in the study will be scheduled for a 1-hour virtual video training session with the research coordinator prior to admission to undergo the transplant. Based on our experience, these training sessions will be coordinated with other clinic appointments at the hospital in efforts to minimize the burden on the caregiver and patient. Once the intervention has begun, the research coordinator will meet with the participants (caregiver-patient dyad) weekly (virtually) to review any questions or concerns and to ensure adherence to the protocol. HSCT patients are typically inpatients for approximately 4 weeks. During this period, the research coordinator will be available by pager and will schedule weekly visits (virtual or in person if needed) with the participants. Once discharged, HSCT patients return to the clinic on a weekly visit in the ambulatory care setting. The research coordinator will meet with participants during these routine appointments or virtually if possible.

#### Fidelity

Based on our prior work, it is critical that adherence and fidelity of the protocol are maintained. Thus, our research staff will develop Intervention Fidelity Guidelines (Multimedia Appendix 3) for the study team to follow and monitor participants, which will help to increase confidence that “study outcomes are due to the intervention being investigated and not due to variability in intervention implementation” [50]. Moreover, the Intervention Fidelity Guidelines will assist in future implementation and dissemination efforts. A rigorous Recruitment and Retention Plan has also been developed and will be adhered to according to good clinical practice (Multimedia Appendix 4).

#### Power Calculation

The power calculation was completed using simulations in the statistical package R. We will enroll 166 caregivers and their care recipients (332 total individuals). Our primary endpoint (ie, caregiver health-related quality of life from the PROMIS Global Health scale) is focused solely on this outcome in caregivers, whereas our secondary endpoints are based on outcomes for both caregivers and patients. Accordingly, we determined the sample size based solely on caregivers. The age- and sex-adjusted norm for the Global Health scale is 50 points, with a standard deviation of 10 points; these statistics have been shown to be generalizable to the HSCT population. With 67 caregivers enrolled in each arm, our study will have power of 0.80, assuming a two-sided type I error rate of 0.05, to detect an effect size of 0.5 between the intervention and control arms. A mean difference of one-half a standard deviation is biologically meaningful and is considered a medium effect size for clinical trials. We will accrue a total of 83 caregivers in each arm to account for dropout, assuming a 5% failure to undergo HSCT, 8% death before day 120, and 10% missing day 120 patient-reported outcomes from participants.

#### Data Analysis

PROMIS measures are based on the item response theory [51], a family of statistical models that link individual questions to a presumed underlying trait or concept of global health represented by all items in the scale. PROMIS instruments are scored using item-level calibrations. The most accurate way to score a PROMIS instrument is to use the HealthMeasures Scoring Service, which our study team has prior experience with. This method of scoring uses “response pattern scoring,” which scores the responses to each item for each participant. Response pattern scoring is especially useful when there are missing data (ie, a respondent skipped an item, or different groups of participants responded to different items).

#### Visit Schedule (Occurring and Planned Visits)

The study intervention period includes the time of admission to the transplant unit through day 120 post-transplant. The average length of hospitalization is 4 weeks. The research coordinator will meet with study participants once weekly during the inpatient hospitalization period. Following discharge, the research coordinator will continue meeting with study participants during routine, standard-of-care weekly visits through day 120 post-transplant (study endpoint). Baseline, day 30, and day 120 post-transplant survey instruments will be completed by study participants.

### Data and Safety Monitoring

This study will be monitored in accordance with the University of Michigan Data and Safety Monitoring Plan for HSCT-specific study protocols. The study team will meet every 6 months or more frequently depending on the activity of the protocol. The discussion will include matters related to the safety of study participants (adverse event and severe adverse event reporting), validity and integrity of the data, enrollment rate relative to expectations, characteristics of participants, retention of participants, adherence to the protocol (potential or real protocol deviations), and data completeness. At these regular meetings, the protocol-specific Data and Safety Monitoring Report form will be completed and signed by the principal investigator or by one of the coinvestigators. Data and Safety Monitoring Reports will be submitted to the University of Michigan Data and Safety Monitoring Committee and the IRB every 6 months for independent review.

## Results

The study protocol complies with the Declaration of Helsinki. This protocol has been approved by the IRB at the University of Michigan and has been registered at ClinicalTrials.gov (NCT04094844).

Due to the current COVID-19 pandemic [52,53], the study has been briefly put on hold. However, recruitment began in August 2020. We have converted all recruitment, enrollment, and onboarding processes to be conducted remotely through video telehealth. Consent will be obtained electronically through the Roadmap 2.0 app.

## Discussion

Caregiver burden is defined as a “negative reaction to the impact of providing care on the caregiver’s social, occupational, and personal roles” [54]. To date, substantial focus has been placed on the wide range of *negative* implications associated with caregiving [55] (eg, depression, anxiety) [56]. Nevertheless, a majority of caregivers have recognized the benefits of caregiving [57,58]. The imbalance of focusing primarily on negative aspects may limit our ability to develop new assessment and intervention methods [59]. Thus, a “corrective focus” is needed in caregiving research to expand knowledge on the *positive* aspects of caregiving [60,61]. Research on self-management suggests that self-efficacy, a positive aspect, can promote caregiver health, well-being, and positive health behaviors (ie, improved sleep and physical activity) [62,63].

The positive aspects of caregiving, such as self-efficacy and positive attitudes toward the caregiver role, may explain how caregivers can positively engage patients in self-care activities [64]. Caregivers with better self-efficacy and well-being (eg, health-related quality of life) may positively impact patients’ health outcomes [65-67]. Simple strategies aimed at enhancing positive thoughts, emotions, and behaviors have been shown to be effective and highly scalable [35-37]. Positive activity interventions such as daily positive reflection, gratitude journals, and conducting acts of kindness have been used in the context of heart disease, cancer, diabetes, and chronic pain [68-73]. Our preliminary data suggest that HSCT caregivers desire these activities to reduce stress and improve well-being.

Caregivers experience significant stress, anxiety, and poor sleep that lead to physiological changes [74]. Indeed, long-term caregiving has been associated with increased physical morbidity [75]. Although caregiver interventions have been shown to reduce emotional distress and increase well-being [76], less is known about the impact of physiological changes on caregiver health and well-being [14]. Previous caregiver assessments have relied on snapshot self-report measures (ie, patient-reported outcomes) [77]. Recent advances in wearable sensors facilitate the noninvasive collection of continuous, real-time measures (eg, sleep, physical activity). These highly time-resolved, objective parameters correlated with subjective patient-reported outcomes may help us to further identify the mechanism of action of an intervention. Further, newer data science techniques may enable data patterns, relationships, and interpretation in ways that were not previously possible [78].

Emerging literature on family caregivers suggests a wide range of health information technology studies that are being conducted [79-82], which have been embraced by both caregivers and patients [16]. We have described the design and research protocol of an mHealth app for HSCT caregivers and patients, which will be tested in a Roadmap 2.0 mobile randomized trial. We outlined the multicomponent features of the mHealth app, study design, inclusion/exclusion criteria, how the intervention will work, recruitment and retention, intervention fidelity guidelines, and data safety monitoring plans. Our multicomponent intervention is innovative, and our mHealth app could have significant impact and wide-ranging clinical applications. Alternatively, even if the primary endpoint of the intervention is not met, we will be able to use the caregiver and patient experiences and secondary outcome findings to create new hypotheses and explore alternative strategies. We are excited about the large multiparameter dataset that will be collected from diverse sources, including caregiver- and patient-reported outcomes, interviews, and physiological markers. Thus, the data streams of Roadmap 2.0 have the potential to provide new understanding about what component of the intervention is driving a desired or undesired outcome. Furthermore, this study has potential to generate new insights into the relationship between caregiver burden, well-being, and health without the dependence on expert trainers, intensive training, or monitoring given its remote features. Ultimately, we anticipate that this study will inform more personalized interventions for caregivers and patients in the future, and address gaps in previous interventions that required face-to-face interactions.

## Appendix

Multimedia Appendix 1 Definitions of multicomponent features of Roadmap 2.0.

Multimedia Appendix 2 Caregiver- and patient-reported outcome measures.

Multimedia Appendix 3 Intervention Fidelity Guidelines.

Multimedia Appendix 4 Recruitment and Retention Plan.

## Abbreviations

HSCT: hematopoietic stem cell transplantation
IRB: institutional review board
mHealth: mobile health
PROMIS: Patient-Reported Outcomes Measurement Information System
