# Supplementary material for: Promoting Health and Well-Being Through Mobile Health Technology (Roadmap 2.0) in Family Caregivers and Patients Undergoing Hematopoietic Stem Cell Transplantation: Protocol for the Development of a Mobile Randomized Controlled Trial
Source: JMIR Res Protoc. 2020 Sep 18;9(9):e19288. doi: 10.2196/19288 (PMC7532463; doi:10.2196/19288)
Supplement: Multimedia Appendix 1 [file resprot_v9i9e19288_app1.docx]

**Multimedia Appendix 1:** Roadmap 2.0 App Multi-Component Features Defined

For study participants randomized to the Treatment Arm they will have access to components A – D outlined below.

Study participants randomized to the Control Arm (defined as all informational or educational resources provided through verbal communication or written hand-out materials) will only have access to component D, outlined below.

All study participants will respond to survey questionnaires on their mobile devices.

1. Positive Activity Exercises (Sample Landing Screen page):
2. Positive Piggy Bank
3. Gratitude Diary
4. Savoring
5. Pleasant Activity Scheduling
6. Random Acts of Kindness
7. Signature Strengths
8. Love Letter
9. Engaging with Beauty


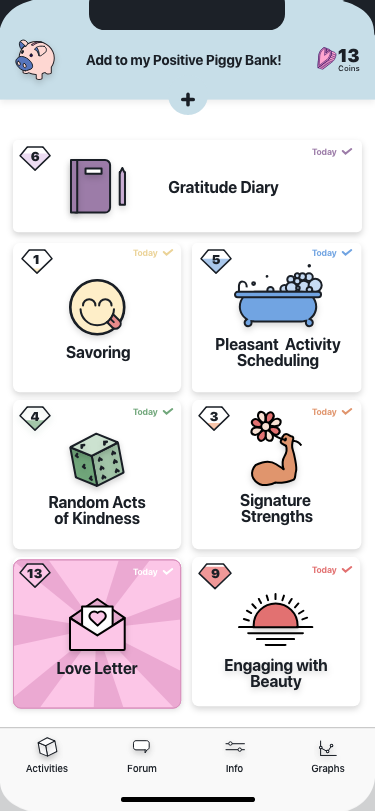


Sample text with instructions provided to the caregiver:

1. Positive Piggy Bank:

As human beings, we tend to focus on negative things, people and events. This focus on the negative can undermine our happiness. Keeping a Positive Piggy Bank can help us focus on all the good things in our world, too.

Step 1: When you observe something that makes you happy, take a moment as savor it. Think about what makes this so special to you.

Step 2: Make a note to capture this thing or moment with enough detail that you can immediately recall what happened later.

Step 3: Now, tap the coin and it will drop into your positive piggy bank.

Step 4: You can make as many of these happy memory “deposits” as you like. The best part is that when you need a little pick-me-up, you may “break” open your piggy bank and read all of these happy notes.

*Examples:*

*My best friend called to cheer me up. It worked!*

*Peach pie with homemade whipped cream, yummy.*

*My husband paid me the kindest compliment today.*

*I love the feel of grass under my feet.*

1. Gratitude Diary:

Feeling grateful is a powerful way to ward off depression and inspire feelings of optimism. It is perhaps the easiest positive emotion to tap in to when things are difficult. For that reason, we encourage you to keep a gratitude diary. You can do that right here! This is how to go about it:

Step 1: Every day, note at least 2 things for which you are grateful. It can be anything – your friends and family, your pets, feeling the sunshine on your face, happy that a friend phoned, receiving a present, being able to take a walk, chocolate cupcakes … anything. Evenings, right before you go to sleep, usually works best.

Step 2: Make a commitment to yourself that you will note at least 2 things every day, but here is a twist - the things you list MUST be DIFFERENT. Try never to repeat anything.

Step 3: Smile as you write these things down. This will help you to feel even more grateful.

*There are examples within the instructions above, but here are some if more are needed:*

*Bigger things: My family*

*My health*

*Having enough food to eat*

*My dear friend, Terry*

*Smaller things: Children laughing*

*The beach*

*Peanut butter*

*Puppies!*

1. Savoring:

Savoring involves recognizing special moments and taking efforts to make them last and be more memorable. You can savor food, experiences, moments with loved ones, anything that brings you pleasure.

Step 1: Consider a typical weekday. Review your morning routine, your daily activities, and your evening rituals, and consider how much time you spend noticing and enjoying the pleasures of the day, both small and large.

Step 2: Every day for the next week, be sure to savor at least two experiences (for example, your morning coffee, or the sun on your face as you walk to your car). Spend at least 2-3 minutes savoring each experience.

Step 3: Log these savoring experiences here so you can revisit them later.

*Here are some more things you could savor:*

Sunsets

Fresh baked cookies

Time with your best friend

Visiting a new place

Playing your favorite game

A walk on a lovely day

1. Pleasant Activity Scheduling:

Providing care for loved ones can be incredibly time consuming. You might have already noticed that you have stopped doing many of the fun things you used to do. Yet, these pleasant activities are incredibly important and can help you better cope with stress. By scheduling and taking part in pleasant activities, you may find that you feel happier and have more energy.

Step 1: Identify activities that you find to be pleasant. These activities do not have to be expensive or time consuming – they just need to be things you enjoy. Activities could include taking a walk in the park, listening to music, working on your hobby, seeing a movie with a friend or reading a great book.

Step 2: Set aside time in the next week to do at least two of these activities. Put them on your calendar like an appointment and treat them with the same importance as you would a doctor’s appointment.

Step 3: Log what you did for your pleasant activity. Have fun, it’s good for you!

1. Random Acts of Kindness:

Although we do kind things daily, we often do not set out to intentionally do something nice for somebody else. Kindness is something always available for us to both give and receive.

Step 1: For this activity, one day this week, do five kind acts all in one day. Take a little time to plan what you are going to do. For the first ***four*** acts, do these for other people. These people can be complete strangers or friends and family members. These can be small acts of kindness such as holding a door open, sharing a genuine compliment or giving somebody a hug.

Step 2: You must also do ***one*** kind thing for you. People who take care of others tend to put them first and forget to be kind to themselves. It's important to take care of yourself, too! Perhaps, you could take a long bubble bath, go for a walk in the park, enjoy a Popsicle or sleep an extra 20 minutes.

Step 3: Smile as you do these kind acts. You are putting good into the world!

*Here are some more ideas about kind acts you could do*:

Leave inspiring message sticky notes to be found.

Give a friend a small gift for no particular reason.

Call a family member out of the blue and tell them how much they mean to you.

1. Signature Strengths:

Character strengths are connected with resilience and buffer people from vulnerabilities that can lead to depression and anxiety. Your unique set of character strengths make you, you. Using these strengths more regularly and in different ways can help you lead a more successful and rewarding life.

Step 1: Based on the Brief Strengths Test, note your top seven strengths.

Step 2: Every day for the next week, use one of these strengths in a way that you have not used it before.

Step 3: Each night, note how you used one of your strengths that day, including what strength you used, how you felt before, during, and after the activity, and whether you plan to repeat it in the future.

*Examples:*

*If “creativity” is one of your strengths and you usually use creativity for arts and crafts, try using creativity to solve a difficult problem at work.*

*If “zest” is a strength of yours and you focus this on your hobby of cooking, try using zest to improve the quality of your friendships.*

*If “hope” is a strength of your and you tend to use hope to strengthen your faith, perhaps you could use hope in your exercise regime to envision the positive strides you can make.*

1. Love Letter:

Finding ways to express warmth, care, deep positive regard, and authentic appreciation to those we love is important to us (the giver) to express, and for the receiver to hear and experience.

Step 1: Think about the love you have for the person for whom you are providing care.

Step 2: Write a brief love letter to this person. In the letter, tell your loved one about your love for him or her, offering your thoughts, feelings and specific examples. Also, consider linking your love to something that happened today or recently.

Step 3: Share your letter with the person you care for.

1. Engaging with Beauty:

Beauty in nature can inspire the emotion of ‘awe,’ beauty in art and skill can inspire admiration, and the witnessing of beauty in positive acts of human behavior can inspire more positive acts echoing like a ripple in a pond.

Step 1: Create a Beauty Log where you will add your observations about three different types of beauty: beauty in nature, beauty that is man-made (e.g., art, music, dance, architecture) or beautiful human behavior (e.g., kind acts, brave acts).

Step 2: Look for beauty as you go through the day. When you observe something that is beautiful, add it to your log in text or photo form.

1. Forum for Caregivers to post positive comments based on Positive Activity exercises that are shared with other Caregiver participants:
   1. Gratitude Room
   2. Acts of Kindness Room
   3. Observed Beauty Room
   4. Strengths Room
   5. Love Letter Room
2. Caregiver-Specific Resources (Guides)
   1. Healthy Nutrition, Physical Activity, and Sleep Tips
   2. Ann Arbor Guide and Caregiver Resource Links
   3. Caregiver’s Guide to Patient’s Symptom Management Tips
   4. Navigating through Hematopoietic Cell Transplantation (HCT): A Guide for Pediatric HCT
3. Mood, Sleep, and Steps
   1. Patients and caregivers will be provided graphs of daily mood, sleep, and steps data (sample data plot shown on next page), depending on whether the participants respond to the daily mood questionnaire – scale between 1-10, anchored to “worst possible” and “best possible,” respectively – and wear the Fitbit Charge 3.
