# Supplementary material for: Promoting Health and Well-Being Through Mobile Health Technology (Roadmap 2.0) in Family Caregivers and Patients Undergoing Hematopoietic Stem Cell Transplantation: Protocol for the Development of a Mobile Randomized Controlled Trial
Source: JMIR Res Protoc. 2020 Sep 18;9(9):e19288. doi: 10.2196/19288 (PMC7532463; doi:10.2196/19288)
Supplement: Multimedia Appendix 2 [file resprot_v9i9e19288_app2.docx]

**Multimedia Appendix 2:** Caregiver- and Patient-Reported Outcome Measures


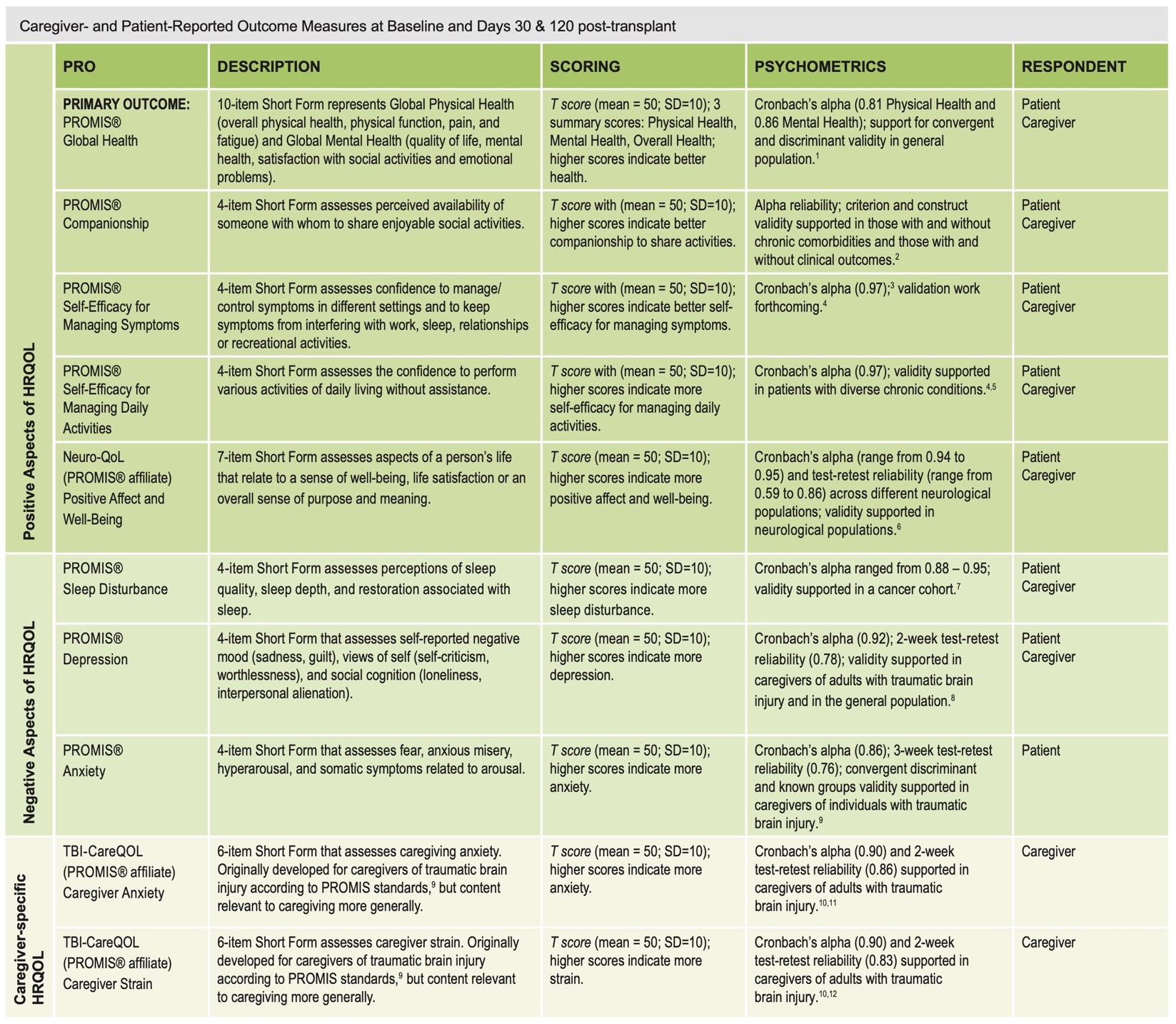


Caregiver-specific health related quality of life (HRQOL): TBI-CareQOL (PROMIS® affiliate) Caregiver Anxiety and Caregiver Strain were both developed by Noelle Carlozzi PhD in caregivers of traumatic brain injury patients. These measures will be examined in caregivers of hematopoietic stem cell transplant patients.

**REFERENCES**

1. Hays RD, Bjorner JB, Revicki DA, Spritzer KL, Cella D. Development of physical and mental health summary scores from the patient-reported outcomes measurement information system (PROMIS) global items. Quality of life research: an international journal of quality of life aspects of treatment, care and rehabilitation. 2009;18(7):873-80.
2. PROMIS, Patient-Reported Outcomes Measurement Information System (HealthMeasures). U.S. Department of Health and Human Services. Scoring PROMIS Companionship Short Form v2.0. October 2014. Last accessed on April 05, 2020.
3. Hahn EA, DeWalt DA, Bode RK, Garcia SF, DeVellis RF, Correia H, et al. New English and Spanish social health measures will facilitate evaluating health determinants. Health psychology: official journal of the Division of Health Psychology, American Psychological Association. 2014;33(5):490-9.
4. Hong I, Velozo CA, Li CY, Romero S, Gruber-Baldini AL, Shulman LM. Assessment of the psychometrics of a PROMIS item bank: self-efficacy for managing daily activities. Quality of life research: an international journal of quality of life aspects of treatment, care and rehabilitation. 2016;25(9):2221-32.
5. PROMIS. Self-Efficacy for Managing Symptoms. <http://www.healthmeasures.net/administrator/components/com_instruments/uploads/PROMIS%20Bank%20v1.0%20-%20Self-Effic-ManagSymptoms_8-5-16.pdf>. Last access on April 05, 2020.
6. Salsman JM, Victorson D, Choi SW, Peterman AH, Heinemann AW, Nowinski C, et al. Development and validation of the positive affect and well-being scale for the neurology quality of life (Neuro-QOL) measurement system. Quality of life research: an international journal of quality of life aspects of treatment, care and rehabilitation. 2013;22(9):2569-80.
7. Jensen RE, King-Kallimanis BL, Sexton E, Reeve BB, Moinpour CM, Potosky AL, et al. Measurement properties of PROMIS Sleep Disturbance short forms in a large, ethnically diverse cancer cohort. Psychological Test and Assessment Modeling, 2016; 58(2):353-70.
8. Schalet BD, Pilkonis PA, Yu L, Dodds N, Johnston KL, Yount S, et al. Clinical validity of PROMIS Depression, Anxiety, and Anger across diverse clinical samples. J Clin Epidemiol. 2016; 73:119-27.
9. PROMIS, Patient-Reported Outcomes Measurement Information System (HealthMeasures). U.S. Department of Health and Human Services. Instrument Development and Psychometric Evaluation; Scientific Standards, May 2012. Last accessed on April 05, 2020.
10. Carlozzi NE, Kallen MA, Hanks, R, Hahn EA, Brickell TA, Lange RT, French LM, Kratz AL, Tulsky DS, Cella D, Miner JA, Ianni PA, Sander AM. The TBI-CareQOL Measurement System: Development and Preliminary Validation of health-Related Quality of Life Measures for Caregivers of Civilians and Service Members/Veterans with Trauamatic Brain Injury. Arch Phys Med Rehabil. 2019; 100(4S):S1-S2.
11. Carlozzi NE, Kallen MA, Sander AM, Brickell Ta, Lange RT, French LM, Ianni PA, Miner JA, Hanks R. The Development of a New Computer adaptive Test to evaluate Anxiety in Caregivers of Individuals with Traumatic Brain Injury: TBI-CareQOL Caregiver-Specific Anxiety. *Arch Phys Med Rehabil* 2019, 100(4S):S22-S30.
12. Carlozzi NE, Kallen MA, Ianni PA, Hahn EA, French lm, Lange RT, Brickell TA, Hanks R, Sander AM. The Development of a New Computer-Adaptive Test to Evaluate Strain in Caregivers of Individuals with TBI: TBI-CareQOL Caregiver Strain. *Arch Phys Med Rehabil* 2019, 100(4S):S13-S21.
