# Supplementary material for: Promoting Health and Well-Being Through Mobile Health Technology (Roadmap 2.0) in Family Caregivers and Patients Undergoing Hematopoietic Stem Cell Transplantation: Protocol for the Development of a Mobile Randomized Controlled Trial
Source: JMIR Res Protoc. 2020 Sep 18;9(9):e19288. doi: 10.2196/19288 (PMC7532463; doi:10.2196/19288)

**Multimedia Appendix 3:** Recruitment and Retention Plan – *Development of Intervention Fidelity Guidelines*


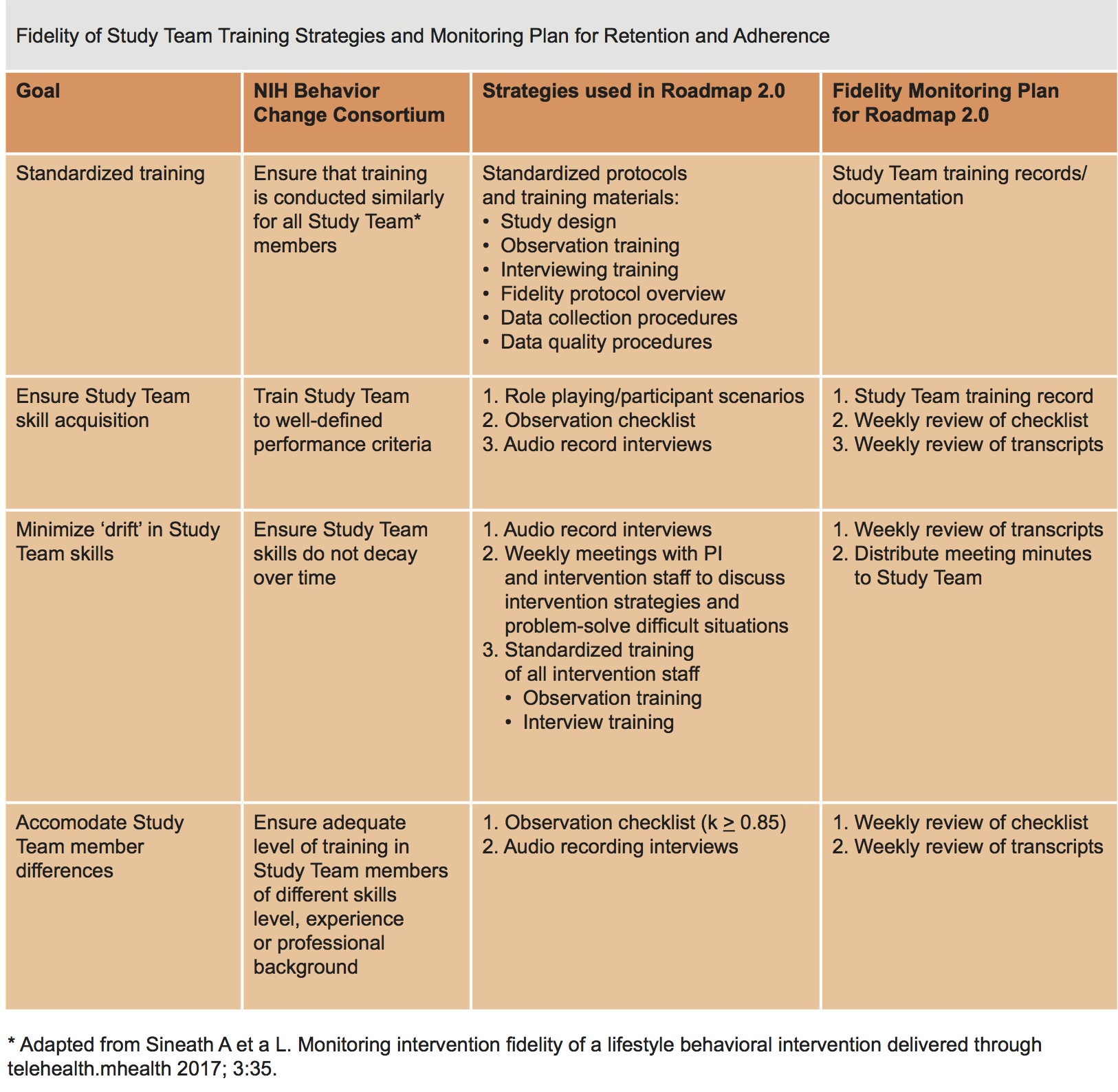

Supplement: Multimedia Appendix 3 [file resprot_v9i9e19288_app3.docx]
