# Supplementary material for: Promoting Health and Well-Being Through Mobile Health Technology (Roadmap 2.0) in Family Caregivers and Patients Undergoing Hematopoietic Stem Cell Transplantation: Protocol for the Development of a Mobile Randomized Controlled Trial
Source: JMIR Res Protoc. 2020 Sep 18;9(9):e19288. doi: 10.2196/19288 (PMC7532463; doi:10.2196/19288)
Supplement: Multimedia Appendix 4 [file resprot_v9i9e19288_app4.docx]

**Multimedia Appendix 4:** Recruitment and Retention Plan

Based on our prior work, we have found the following strategies to be effective and low burden in recruiting potential study participants:

1. Study participants will be recruited through the University of Michigan Blood and Marrow Transplantation (BMT) Program by BMT physicians and nurse coordinators. The BMT Program conducts weekly, Multidisciplinary BMT Patient Meetings where potential patients being considered for future hematopoietic stem cell transplantation (HSCT), as well as patients actively undergoing BMT and patients re-admitted with transplant-related complications are reviewed and discussed. The meeting participants include BMT physicians, RN coordinators, social workers, advanced practitioners, pharmacists, hematology-oncology fellow trainees, billing/insurance representatives, research coordinators, and research assistants. During the meeting, BMT patients are evaluated and considered for eligibility on all IRB-approved clinical trials/research studies. Approximately 98% of our patients enroll onto clinical research studies.
2. The BMT Program has maintained a unique BMT Clinical Research Biorepository since 2000 that contains detailed clinical information with over 200 data variables per patient and their associated biospecimens (e.g., blood, urine, stool), stored in accordance with our IRB-approved protocol. The database contains thousands of individuals who have registered to be contacted about future study opportunities.
3. The University of Michigan health system’s electronic health record (EHR) is based on the Epic software (MiChart), which allows researchers, with IRB approval, to contact (via email, postcards, or in-person) individuals who meet specific criteria and may be eligible to participate in the study. For example, with IRB-approval, the Study Team can access the daily Inpatient and Outpatient Lists, which will enable them to identify potential participants and meet them in-person during a corresponding patient encounter.
4. With IRB-approval, we will distribute flyers in the BMT Unit (Outpatient BMT Clinic) to inform potential individuals about the study. We will also distribute emails to potential study participants (e.g., BMT health care providers, non-BMT health care professionals, community engagement partners).
5. The Michigan Institute for Clinical and Health Research, supported by the National Institutes of Health Clinical and Translational Science Award Program, will assist our team with developing and reviewing recruitment materials, and identifying obstacles that may affect participant recruitment and retention.

Retention is important to the fidelity of this mobile randomized trial. Once the patient is enrolled in the study, our goal is to provide a positive, rewarding experience for the caregiver and patient – help them feel comfortable, respected, and well-informed. In efforts to minimize the risk of study drop-out, we will:

1. Maintain strong communication with the participants over the duration of the study, the Research Coordinator will touch base with the caregiver/patient at least weekly during the inpatient phase of treatment and then at each outpatient clinic visit. We have used this strategy for all of our other clinical studies and have found that this approach is very useful to the patient/family and also in enhancing retention. This helps the Study Team to remain engaged with the patient/family throughout the study period.
2. Provide the patient/family with a personal notebook/folder containing an IRB-approved Study Calendar. The caregiver/patient can use the notebook to write down any immediate thoughts or concerns to then share at their weekly clinic visits. The Research Coordinator will review the diary and/or notes with the patient/family at each of these visits. This will help us to better understand protocol adherence and what concerns he/she may be experiencing.
3. Incorporate the caregiver/patient voice during each visit (i.e., feelings and attitudes toward study participation). We will acknowledge their efforts and contribution to research.
4. With IRB-approval, participants will be compensated for their time. Participants will be compensated for each completed survey questionnaire ($25 per time-point), or up to $75 for completing all three time-points (baseline, day 30 and day 120 post-transplant). In addition, participants will get to keep the Fitbit Charge 3.
5. Track and monitor our efforts – we will monitor the target number of patients (recruitment, enrollment), how many have enrolled, how many have dropped out/discontinued before the target date (and reasons why, follow up with the subject), fidelity/adherence, and how many have completed the study. We will review these data during our weekly Investigator’s Meeting and the Multidisciplinary BMT Patient Meeting. Updates will also be reviewed and discussed at the BMT Data Safety and Monitoring Committee meeting.
6. Develop and Implement an Intervention Fidelity Guidelines for all of the Research Staff to adhere to (Multimedia Appendix 3: Intervention Fidelity Guidelines). The Study Team has experience in developing and implementing similar protocols in their research program.
7. Develop an informational website for study participants (<http://Roadmap.Study>).
